# An integrative pharmacology-based study on the pharmacological activity and mechanism of xiaoji-chenpi formula (XCF) against MAFLD

**DOI:** 10.3389/fphar.2025.1521111

**Published:** 2025-03-07

**Authors:** Shufei Liang, Yang Dong, Zukang Chang, Pingping Guo, Jinghan Jia, Gangao Yang, Yongning Chen, Ling Dong, Xiaoxue Xu, Tianqi Cai, Tianxing Li, Yini Fang, Wenlong Sun, Lingru Li, Chao Wang, Xinhua Song

**Affiliations:** ^1^ School of Life Sciences and Medicine, Shandong University of Technology, Zibo, Shandong, China; ^2^ National Institute of TCM Constitution and Preventive Medicine, Beijing University of Chinese Medicine, Beijing, China; ^3^ Monitoring and Statistical Research Center, National Administration of Traditional Chinese Medicine, Beijing, China

**Keywords:** metabolic-associated fatty liver disease, xiaoji-chenpi formula, zebrafish, RNA-seq, INSIG1/SREBP1 pathway

## Abstract

Metabolic-associated fatty liver disease (MAFLD) is a common chronic metabolic disease worldwide that seriously threatens human health. The Xiaoji-chenpi formula (XCF), derived from QingGanSan (QGS), has previously been proven to be clinically effective in MAFLD. However, its pharmacological activity and mechanism have not been studied in depth. In this study, we explored and determined the optimal amounts of cholesterol and fat additives (4% and 20%, respectively) for the modeling of zebrafish MAFLD via orthogonal tests. The zebrafish MAFLD model was used for preliminary screening and determination of the pharmacological activity of XCF on MAFLD. XCF significantly reduced the body mass index (BMI), improved the morphology of liver cells and reduced the number of lipid vacuoles, which were better than the corresponding pharmacological activity of silymarin and resveratrol in zebrafish with MAFLD. The four main active compounds in XCF were identified by HPLC analysis as chlorogenic acid, naringin, hesperidin and quercetin. MAFLD in the mouse model was induced by a high-fat diet (HFD), and the pharmacological activity and mechanism of XCF were investigated by measuring plasma and hepatic physiological indices. XCF reduced the plasma TC and TG levels, reduced the liver TC and TG levels, and relieved liver lipid accumulation and inflammation in the mice. Key differentially expressed genes were identified through transcriptomics and detected via western blotting. XCF regulated the levels of INSIG1, SREBP1, FASN, ACC, SPP1, LGALS3, TNF-α and IL-1β in the livers of the MAFLD mice and improved the disease status. Our research provides a basis for developing an effective functional product for treating the occurrence and progression of MAFLD.

## 1 Introduction

Nonalcoholic fatty liver disease (NAFLD), renamed metabolic-associated fatty liver disease (MAFLD), has become a common chronic liver disease in most parts of the world and is associated with excessive lipid accumulation and lipid metabolism imbalance in the liver (Eslam et al., 2020). Excessive lipid accumulation disrupts the ability of the liver to metabolize lipids, leading to increased oxidative stress, inflammation, and even fibrosis (Browning and Jay, 2004; Geng et al., 2021). Changing unhealthy lifestyles, rationally adjusting the dietary structure, and losing weight are effective ways to treat this disease (Friedman et al., 2018). However, for most patients, maintaining body weight within the ideal range for a long period is difficult. Therefore, corresponding functional products and drugs are still needed for adjuvant treatment.

In recent years, traditional Chinese medicine (TCM) and natural products have been widely used to treat MAFLD, liver fibrosis, and other conditions (Dai et al., 2021; Li W.-Q. et al., 2022; Zhu et al., 2023). TCM has multiple targets and pathways for reducing liver lipid deposition and exerts anti-inflammatory and antifibrotic pharmacological activity. Based on the overall concept of traditional Chinese medicine and the core concept of dialectical treatment, traditional Chinese medicine prescription is the key intervention method in the clinical practice of traditional Chinese medicine. The principle of “Jun-Chen-Zuo-Shi” is an important guiding principle for formulating and optimizing traditional Chinese medicine prescriptions, which explains the primary and secondary relationship of herbs compatibility and medication principles in prescriptions, thus ensuring the pertinence and effectiveness of prescriptions (Qiu, 2007). Disassembly of prescriptions is an important link in the research and practice of traditional Chinese medicine. It is not a simple addition or subtraction of herbs, but needs to optimize the composition and pharmacological activity of prescriptions on the basis of maintaining the overall pharmacological activity of prescriptions, so as to achieve better collaborative pharmacological activity. It is helpful to guide clinical medication and individualized treatment.

Our research group previously developed a series of Chinese herbal prescriptions that can improve MAFLD, including QGS and its dismantled formulas. QGS composed of six herbs, including *Polygonatum sibiricum* Delar. ex Redoute [Asparagaceae; Polygonatum sibiricum radix et rhizoma], *M. alba* L. [Moraceae; Morus alba leaves], Cichorium intybus L. [Asteraceae; Dry aboveground parts or roots of Cichorium intybus], *Cirsium setosum* (Willd.) MB. [Asteraceae; Dry aboveground part of Cirsium setosum (Willd.) MB], *Citrus reticulata* Blanco [Rutaceae; Citrus reticulata pericarpium] and Glycyrrhiza uralensis Fisch. ex DC. [Fabaceae; Glycyrrhizae radix et rhizoma], at a ratio of 15:10:10:10:6:6, has been proven to have good therapeutic effects on MAFLD in our previous studies (Yang et al., 2021). For the treatment of MAFLD, the formula of QGS was dismantled to obtain XCF (Xiaoji-Chenpi formula, which is composed of *C*. *setosum*, the peel of *C*. *reticulata* and *Morus alba* leaves, at a ratio of 5:3:5) and XJF (Xiaoji-Jujv formula, which is composed of *C*. *setosum*, *C*. *intybus* and *M. alba* leaves, at a ratio of 1:1:1) on the basis of the basic theory of traditional Chinese medicine. Dismantled formulas of QGS, XCF and XJF also have certain effects in clinical practice. In our previous research (patent number: 202110690958.8), it was predicted through network pharmacology that the pharmacological activity of XCF is superior to that of XJF. Although the activities of these formulas have been preliminarily predicted, more experiments are needed to further compare these activities and explore the underlying mechanism.

Although there are multiple studies on traditional Chinese medicine (TCM) formulas, systematic screening and validation methods are lacking. The commonly used classic rodent models have limitations, including high cost and complexity (Kucera and Cervinkova, 2014; Jahn et al., 2019; Flessa et al., 2022). In recent years, the zebrafish has emerged as a potent model for studying lipid metabolism-related liver diseases owing to its small size, low maintenance costs, and high physiological similarity to humans, particularly with respect to cell types, tissue structures, and liver function (Mujica and Hoed, 2023). The use of zebrafish provides an effective strategy for identifying and validating the pharmacological activity and mechanisms of TCM in the MAFLD model.

RNA sequencing technology, which involves high-throughput screening and target identification, has significantly broadened the application of TCM (Lo et al., 2012; Lowe et al., 2017). By analyzing RNA-seq data from cell and tissue samples treated with various drugs, disease-related signaling pathways and key genes can be elucidated. In a recent study on QGS, RNA-seq data analysis identified SREBP1 as a pivotal target gene for alleviating MAFLD (Yang et al., 2021). Further validation via immunofluorescence and western blotting confirmed the capacity of QGS to inhibit SREBP1 activation, thereby ameliorating high-fat diet (HFD)-induced lipid metabolism disorders in rats. This comprehensive approach offers an effective strategy for identifying and validating target genes in TCM research.

In this study, we introduced a zebrafish disease model and systematically explored the optimal conditions for constructing a zebrafish MAFLD model to improve screening efficiency and accuracy. We subsequently validated the pharmacological activity of XCF and XJF in this zebrafish model. HPLC technology was employed to analyze the composition of XCF. Furthermore, a HFD was used to establish a MAFLD mouse model, the effect of XCF ethanol extract on MAFLD mouse was studied, and the potential mechanism was elucidated by combining RNA sequencing and the GeneCards database. Finally, immunoblotting was used to verify the molecular mechanism by which XCF improves MAFLD. Through this study, we developed a standardized method for screening many TCM formulas, which provides a reference for studying the mechanism of TCM.

## 2 Materials and methods

### 2.1 Herbal materials and extracts preparation

The detailed information on the composition of XCF and XJF in terms of scientific, species, pharmaceutical, and Chinese phonetic names and origins can be found in Table 1. For XCF preparation, *C*. *setosum* and *M. alba* leaves and *C*. *reticulata* pericarpium were mixed and crushed at a specific dose (50 g:50 g:30 g). XJF was composed of *C*. *setosum*, *C*. *intybus* and *M. alba* leaves were mixed and crushed at a specific dose (50 g:50 g:50 g). Mixed powderpassed through a sieve of 80 meshes, and then extracted with 75% ethanol aqueous solution (75:25, v/v) at low temperature for 48 h. The ratio of material to liquid was 1:10. The filtrate was subsequently filtered with a cloth funnel. The above operation was repeated twice, all the extracts were combined, a rotary evaporator was used to remove the ethanol, and the ethanol was concentrated to a 1/4 volume (concentrate I). To remove macromolecular polysaccharides to the greatest extent possible, anhydrous ethanol was added to concentrate I, and the final ethanol concentration of the solution was adjusted to 75%. After standing at low temperature for 24 h, the insoluble substances were removed by filtration, and filtrate I was obtained. Then, the mixture was rotated and evaporated to the volume of filtrate I (concentrated liquid II). Next, the oil was removed by petroleum ether solvent extraction via a liquid separation funnel. The lower liquid was then spun to remove the residual petroleum ether to obtain concentrated liquid III. Finally, the formula ethanol extract powder was prepared via vacuum freeze dryer. After drying, XCF extract weight is 10.8 g and the extraction rate is 8.31%. XJF extract weight is 11.3 g and the extraction rate is 7.53%.All the above rotary evaporation operating temperatures were maintained at 55°C–65°C.

**TABLE 1:** The components of XCF and XJF in scientific, species, pharmaceutical, and Chinese Pin Yin names and place of origin.

Scientific name	Species name	Pharmaceutical name	Chinese pin yin	Place of origin (commercial supplier/province)
*Cirsium setosum* (Willd.) MB.	*Cirsium setosum*	Cirsii herba	Xiao ji	Beijing Tongrentang Zibo Pharmacy Chain Co., Ltd., of Zibo, Shandong
*Citrus reticulata* Blanco	*Citrus reticulata*	Citrus reticulata pericarpium	Chen pi	Beijing Tongrentang Zibo Pharmacy Chain Co., Ltd., of Zibo, Shandong
*Morus alba* L	*Morus alba*	Mulberry leaf (leaves)	Sang ye	Beijing Tongrentang Zibo Pharmacy Chain Co., Ltd., of Zibo, Shandong
Cichorium intybus L	*Cichorium intybus*	Chicory	Ju jv	Beijing Tongrentang Zibo Pharmacy Chain Co., Ltd., of Zibo, Shandong

Subsequent operations for *C*. *setosum* extract (CSE), the peel of *C*. *reticulata* extract (CRPE) and *M. alba* leaves extract (MLE) were also carried out according to the above method and process.

### 2.2 Chemical analysis and quality control of XCF

The active compounds contained in XCF were assessed by high-performance liquid chromatography (HPLC) spectrometry. A Shim-pack GIST C18-AQ HPLC column (250 × 4.6 mm, 5 μm; Shimadzu. Ltd., Tokyo, Japan) was used, with the mobile phase comprising A: 100% methanol and B: 0.2% phosphoric acid water. The flow rate was 1.0 mL/min, and the absorption wavelength was 276 nm and 254 nm. The chosen gradients were as follows: 5%–20% A (0–10 min), 20%–25% A (10–15 min), 25%–50% A (15–30 min), 50%–85% A (30–40 min), and 85% A (40–60 min).

Chlorogenic acid (catalog #B20782), naringin (catalog #B21594), hesperidin (catalog #B20182) and quercetin (catalog #B20527) were obtained from Shanghai Yuanye Biological Technology Co., Ltd., (Shanghai, China). Appropriate amounts of chlorogenic acid, naringin, hesperidin and quercetin were accurately weighed and placed in four 10 mL flasks, and the volumes were adjusted to scale with methanol. Standard solutions with mass concentrations of 1.2000 mg/mL, 0.6250 mg/mL, 0.2500 mg/mL, and 1.5000 mg/mL were prepared. The standard solutions were each diluted in half to obtain five standard solutions of different mass concentrations, which were successively injected into a high-performance liquid chromatographic instrument. The standard curves and linear regression equations of the four chemicals were calculated by setting the mass concentration of the commercial standard compounds as X and the peak area of the standard bottle as Y. The contents of the above four standards in the CSE, CRPE, MLE and XCF were also calculated.

Monitor batch-to-batch consistency by analyzing marker compounds using HPLC. Prepare three different batches of XCF using the same preparation method, inject them separately under the same chromatographic conditions, and compare them with commercial standard compounds. Monitor UV absorbance at 312/276/254 nm for fingerprint analysis.

### 2.3 Animals and experimental design

#### 2.3.1 Zebrafish experiments

3–4 months old wild type AB strain female zebrafish purchased from Shanghai FishBio Co., Ltd., were raised under standard laboratory conditions with a 14-h light/10-h dark cycle at a temperature of 28.5°C ± 1°C. The water was replaced daily. Water quality was tested regularly: the dissolved oxygen was >6.0 mg/L, the pH was 7.0–7.2, the nitrogen content was <0.50 mg/L. All experimental diets for zebrafish were customized by SYSE Bio tech. Co., LTD., (Changzhou, China). Detailed dietary information can be obtained from Supplementary Table S1. The experimental grouping of three batches of zebrafish were shown in Supplementary Figure S5. All experimental procedures were conducted in accordance with ethical approval by the Institutional Animal Care and Use Committee of Shandong University of Technology (approval certification number: YLX20200,905).

Experiment 1 High-fat diet-fed adult zebrafish model

After 3-day adaptive feeding, a total of 64 fish were randomly assigned to eight groups. Feed with different fat contents (8%, 18%, 20%, 22%, 24%, 26%, 28%, and 30%) was fed to the zebrafish twice a day with appropriate overfeeding. The entire experiment lasted for 6 weeks.

Experiment 2 The high-cholesterol diet-fed adult zebrafish model

After 3-day adaptive feeding, a total of 56 fish were randomly assigned to seven groups. Feed with different cholesterol and fat contents (0% and 8%, 0% and 20%, 1% and 20%, 2% and 20%, 4% and 20%, 6% and 20%, 8% and 20%) was fed to the zebrafish twice a day with appropriate overfeeding. The entire experiment lasted for 6 weeks.

Experiment 3 Screening of formulas using HFCD zebrafish

After 3-day adaptive feeding, a total of 48 fish were randomly assigned to six groups. NC group: 8% fat content feed; HFCD group and other treatment groups: 4% cholesterol and 20% fat content feed. XCF group: the XCF extract treatment group was placed in an aqueous solution of 5 mg/L XCF extract overnight for 12 h per day; XJF group: the XJF extract treatment group was placed in an aqueous solution of 5 mg/L XJF extract overnight for 12 h per day; Resveratrol group: the resveratrol treatment group was placed in an aqueous solution of 5 mg/L resveratrol overnight for 12 h per day; Silymarin group: the silymarin treatment group was placed in an aqueous solution of 5 mg/L silymarin overnight for 12 h per day. Both the NC group and the HFCD group were incubated overnight in an aqueous solution for 12 h per day. The entire experiment lasted for 6 weeks.

After 6 weeks, zebrafish were fasted for 24 h. Then all zebrafish were anesthetized with 0.2% ethyl3-aminobenzoate methanesulfonate (Sigma, catalog #E10521) for 3–5 min and euthanized. After the body surface water was dried, the body weight (g) was measured via an analytical balance, and the length (cm) from the fish mouth to the end of the tail fin was measured with a ruler. The BMI of adult zebrafish was calculated at the end of the feeding experiment via the following formula: BMI = weight/length^2^ (kg/m^2^).

#### 2.3.2 Mouse experiment

Six-week-old male Kunming mice were obtained from the Shandong Laboratory Animal Center (Jinan, China) (approval number SCXK 2021-0003) and housed under standard laboratory conditions (25°C ± 3°C, 55%–65% humidity, and a 12-h light/dark cycle). After 1 week of adaptive feeding, all the mice were randomly divided into three groups: the normal control group (NC group; n = 8, standard diet), high-fat diet group (HFD group; n = 8, high-fat diet), and XCF treatment group (XCF group; n = 8, high-fat diet). The mice in the HFD group and XCF group were fed a high-fat diet. The mice in the NC group remained on the standard diet throughout the whole process. XCF extract powder was stored in sealed dark centrifuge tube at −20°C. An 80 mg/ml XCF aqueous solution was prepared before gavage. The dose was determined according to the conversion equation of the surface area between humans and mice. According to the best practice in phytopharmaceutical research guideline (Heinrich et al., 2020), *in vivo* experiments were conducted based on the recommended safe dosage. The XCF group was given 80 mg/mL XCF extract dry powder aqueous solution (200 mg/kg, equivalent to containing 2.4 g/kg crude drug) daily for 12 consecutive weeks through oral gavage. In contrast, the NC and HFD groups received the same volume of saline solution. The entire experiment lasted for 12 weeks. All experimental diets for mouse were customized by SYSE Bio tech. Co., LTD. Detailed dietary information can be obtained from Supplementary Table S2. The current study protocol followed international ethical guidelines, and all animal handling procedures were performed in a standard laboratory and approved by the Institutional Animal Care and Use Committee of Shandong University of Technology (approval certification number: YLX20200905).

### 2.4 Biochemical measurements

Body weights and food intake were recorded each week. The mice were anesthetized after 12 weeks of feeding. The blood was collected (0.8–1.2 mL per mouse) and then centrifuged (5 min, 4°C, 3000 rpm) to obtain the plasma. Commercial kits, including total cholesterol (TC, catalog #A111-1-1), triglyceride (TG, catalog #A110-1-1), low-density lipoprotein cholesterol (LDL-C, catalog #A113-1-1), high-density lipoprotein cholesterol (HDL-C, catalog #A112-1-1), alanine aminotransferase (ALT, catalog #C009-2-1), and aspartate aminotransferase (AST, catalog #C011-2-1) kits, were obtained from Nanjing Jiancheng Bioengineering Institute (Nanjing, China). Commercial kits were used to measure the levels of TC, TG, LDL-C, HDL-C, AST, and ALT in the plasma. Moreover, the levels of TC and TG in the liver were also measured with commercially available kits. Total protein in hepatic tissue homogenates was measured with a BCA kit.

### 2.5 Histological observations

Mouse liver tissues (n = 3) and adult zebrafish liver tissues (n = 5) from each group were collected and fixed with 4% paraformaldehyde for 24 h. Then, the samples were carefully embedded in paraffin and sectioned into 3-μm-thick sections, which were stained with hematoxylin and eosin (H&E) or Sirius red. All the samples were observed under a 240-W microscope for pathological analysis. The liver lipids (% staining area) were analyzed by ImageJ (https://imagej.nih.gov/ij/). Tissue fixative was purchased from Wuhan Servicebio Technology Co., Ltd., (Wuhan, China).

### 2.6 RNA sequencing analysis

RNA sequencing was conducted using homogenized liver tissue. Two independent experiments were performed as follows: the NC and HFCD treatments in zebrafish and the NC, HFD and XCF treatments in mouse. Three replicates were performed for each group. The RNA extraction and sequencing procedure was performed by Majorbio. Total RNA was extracted via TRIzol reagent (Qiagen, Germany) according to the manufacturer’s instructions. RNA sequence library construction was performed via the Illumina TruSeqTM RNA Sample Prep Kit method. The Illumina NovaSeq 6,000 platform (LC Science, United States) was subsequently used for quantification and sequencing according to a standard sequencing protocol. The raw paired end reads were trimmed and quality controlled by fastq (Version 0.19.5; Source: https://github.com/OpenGene/fastp) with default parameters. Then clean reads were separately aligned to reference genome (Mus_musculus reference version: GRCm38. p6; Source: http://asia.ensembl.org/Mus_musculus/Info/Index; Danio_rerio reference version: GRCz11; Source: http://asia.ensembl.org/Danio_rerio/Info/Index) with orientation mode using HISAT2 software (Version 2.1.0; Source: http://ccb.jhu.edu/software/hisat2/index.shtml). Next, transcript quantification was performed via RSEM (Source: http://deweylab.biostat.wisc.edu/rsem/) with the TPM method to produce read counts. The read counts were determined to generate a gene expression profile with DESeq2 (Source: http://bioconductor.org/packages/stats/bioc/DESeq2/), in which the default filter conditions were used (P-adjust <0.05 and | log_2_ FC | ≥ 1). Visualize and cluster the expression of genes in each sample/group with hierarchical clustering algorithm parameters. Principal Component Analysis (PCA), Venn analysis and KEGG (Source: http://www.genome.jp/kegg/) enrichment analysis were performed via the online platform of the Majorbio Cloud Platform (www.majobio.com). PPI was conducted via the online website STRING (https://cn.string-db.org/).

Raw data were loaded into NCBI with accession PRJNA1180292 (zebrafish) and PRJNA1180519 (mouse).

### 2.7 Western blot analysis

Liver tissues were homogenized with RIPA lysis buffer supplemented with 1% protease and phosphatase inhibitor cocktail (Beyotime, China) to extract total protein. The protein concentration was measured with a BCA kit (Beyotime, China) according to the manufacturer’s instructions. The samples were separated by 8%–12% SDS‒PAGE and then transferred to 0.22/0.45 μm PVDF membranes. The membranes were subsequently incubated with 5% nonfat milk for approximately 2 h for blocking. The primary antibodies were subsequently added, and the membranes were incubated at 4°C overnight. The membranes were washed three times with TBST for 10 min, incubated with secondary antibodies at room temperature for 2 h and then washed again. A variety of specific antibodies, including an IL-1β antibody diluted to 1:1000 (catalog #16806-1-AP, Proteintech), a TNF-α antibody diluted to 1:1000 (catalog #17590-1-AP, Proteintech), an SPP1 antibody diluted to 1:2000 (catalog #30200-1-AP, Proteintech), a LGALS3 antibody diluted to 1:1000 (catalog #14979-1-AP, Proteintech), an INSIG1 antibody diluted to 1:1000 (catalog #55282-1-AP, Proteintech), an SREBP1 antibody diluted to 1:500 (catalog #14088-1-AP, Proteintech), an ACC antibody highly diluted to 1:500 (catalog #A15606, ABclonal) and a FASN antibody highly diluted to 1:500 (catalog #bs-1498R, Bioss), were used in our experiments. The primary antibody against β-actin and the anti-rabbit secondary antibodies diluted to 1:2000 (catalog #20536-1-AP, SA00001-2) were also purchased from Proteintech Co. Ltd. (Wuhan, China).

Three samples were selected for analysis. The protein bands were visualized via an enhanced chemiluminescence (ECL) plus kit (Beyotime, China), and the band densities were quantified via ImageJ software. All protein expression levels were normalized to that of β-actin. All original images have been submitted to the Supplementary Material PDF file (named Uncropped Western Blots not for publication).

### 2.8 Statistical analysis

Statistical analyses were performed with GraphPad Prism 8. All the data were analyzed via one-way ANOVA, and Dunnett’s test was used for *post hoc* analysis. All the data are presented herein as the means ± standard errors of the means (SEMs). The letters (a, b, c, d) represented multiple comparison results among various different dietary groups or different drug treatment groups. The same letters indicated no significant differences, while different letters indicated significant differences in statistical data. Results with p values of <0.05 were considered statistically significant.

## 3 Results

### 3.1 Construction of the zebrafish MAFLD model and transcriptomic analysis

**FIGURE 1:**
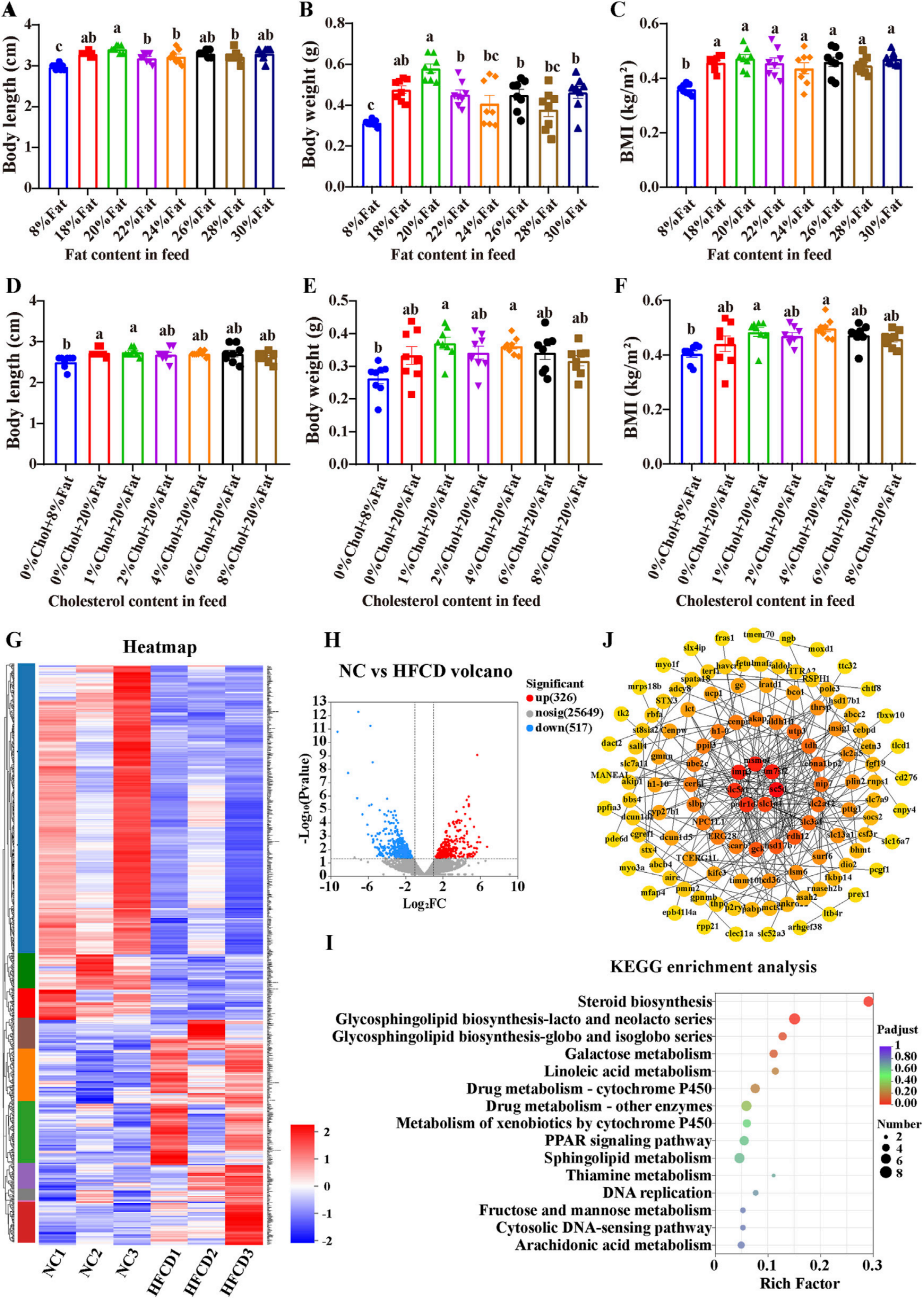
Generation of the zebrafish MAFLD model and transcriptomic analysis. Zebrafish experiment 1: Zebrafish were fed different fat contents: **(A)** Body length, **(B)** Body weight, **(C)** BMI (body mass index); experiment 2: Zebrafish were fed different fat and cholesterol contents: **(D)** Body length; **(E)** Body weight; **(F)** BMI; data are presented as the means ± SEMs (n = 8). Data with different letters are significantly different (P < 0.05). **(G)** Heatmap of differential gene clustering; **(H)** Differential gene volcano plot (NC vs. HFCD); **(I)** KEGG enrichment analysis. **(J)** PPI of core genes related to lipid metabolism. NC: normal control group; HFCD: high-fat and high-cholesterol diet group.

We determined the optimal amount of fat and cholesterol additives that can induce MAFLD in zebrafish via an orthogonal test. First, we carried out an exploration of the optimal amount of fat additive. Eight groups of zebrafish were fed diets with different fat contents (no cholesterol was added to the diet). Compared with that of the 8% fat group, the weight gain of the 20% fat group was greater (P < 0.05, Figures 1A–C). Therefore, we determined that 20% was the optimal concentration for high fat content. We subsequently conducted experiment to determine the optimal cholesterol additive dose. Compared with those of the 8% fat group, the body length, body weight and BMI of the 20% fat group and the 20% fat combined with different concentrations of cholesterol treatment groups were significantly greater, and the BMIs of the 4% cholesterol and 20% fat treatment groups were significantly greater (P < 0.05, Figures 1D–F). Therefore, 4% cholesterol and 20% fat were selected as the optimal feed additive amounts for constructing the zebrafish MAFLD model.

Figure 1G shows the changes in the expression of all differentially expressed genes in different samples from the NC and HFCD groups. Figure 1H shows more intuitively that we detected a total of 843 differentially expressed genes. Compared with the NC group, the HFCD group presented 517 downregulated genes and 326 upregulated genes. As shown in Figure 1I, KEGG analysis revealed that the differentially expressed genes were enriched mainly in the sterol biosynthesis pathway. The above results indicate that high-fat and high-cholesterol diets may induce MAFLD in zebrafish by regulating sterol biosynthesis. To further elucidate the specific genes associated with lipids in the liver transcripts of HFCD, we downloaded a gene set with the keyword ‘Lipid metabolism’ from the GeneCards public database. Venn 2.1 software was used. A total of 149 interlocking genes were obtained from the public database gene set, and the transcriptomes of the differentially expressed genes are shown in Figure 1J.

### 3.2 Effects of different herbal formulas on BMI and morphological changes in liver cells of zebrafish with MAFLD

**FIGURE 2:**
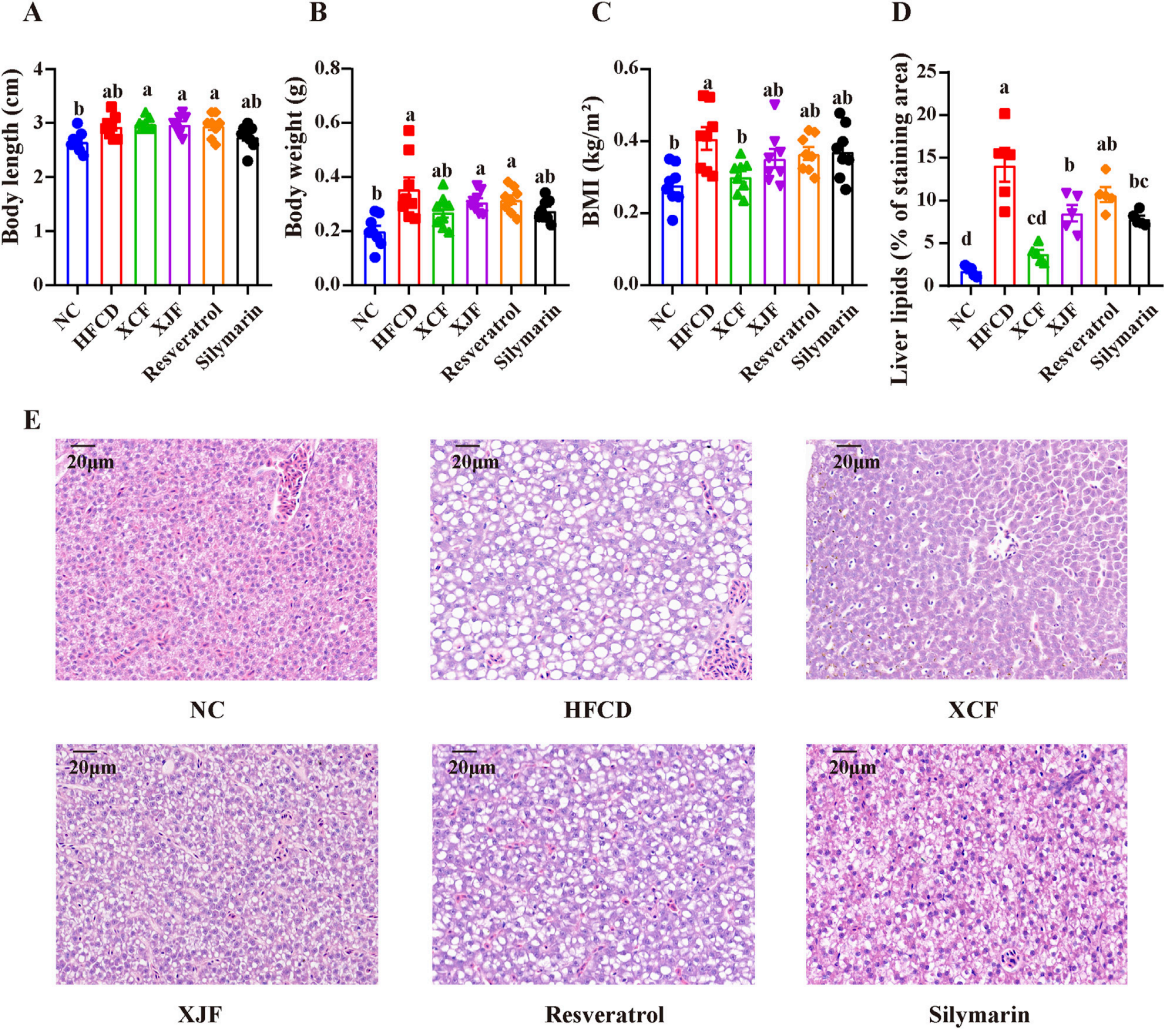
Effects of different herbal formulas on BMI and morphological changes in the liver cells of zebrafish with MAFLD. **(A)** Body length (n = 8); **(B)** Body weight (n = 8); **(C)** BMI (n = 8); **(D)** Liver lipids (% of staining area) (n = 5); **(E)** Micrographs of H&E-stained livers (bar = 20 μm). The data are presented as the means ± SEMs. Data with different letters are significantly different (P < 0.05). NC: normal control group; HFCD: high-fat and high-cholesterol diet group; XCF: xiaoji-chenpi formula extract treatment group; XJF: xiaoji-jujv formula extract treatment group; Resveratrol: resveratrol treatment group; Silymarin: silymarin treatment group.

To further validate the activity of XCF and XJF, resveratrol and silymarin were also used as positive control drugs in the zebrafish experiments. Compared with those in the NC group, the effects of body length changes in the HFCD group were not significant, and the body weights and BMIs increased significantly, which was consistent with the results of the first two gradient treatment experiments (Figures 2A–C). The body weights in all the treatment groups were essentially consistent with those in the HFCD group, with no significant changes. Compared with those of the HFCD group, the body weights and BMIs of all the treatment groups decreased to varying degrees. Among them, only after XCF treatment did the BMIs of zebrafish with MAFLD decrease significantly. H&E staining and quantification of liver lipids revealed that the hepatic structure of the zebrafish in the NC group was normal and that the shape of the liver was full and round. However, hepatic steatosis in the HFCD group was widely distributed in the liver tissue, and many round vacuoles of different sizes were diffuse in the cytoplasm. Compared with that in the HFCD group, the liver cell morphology in the XCF group was effectively restored, and the number of round vacuoles was greatly reduced (Figures 2D, E). These data indicate that XCF administration significantly reduced lipid accumulation in the liver. Liver lesions in the XJF, resveratrol and silymarin groups also improved slightly to varying degrees.

### 3.3 Chemical analysis of XCF by HPLC

**FIGURE 3:**
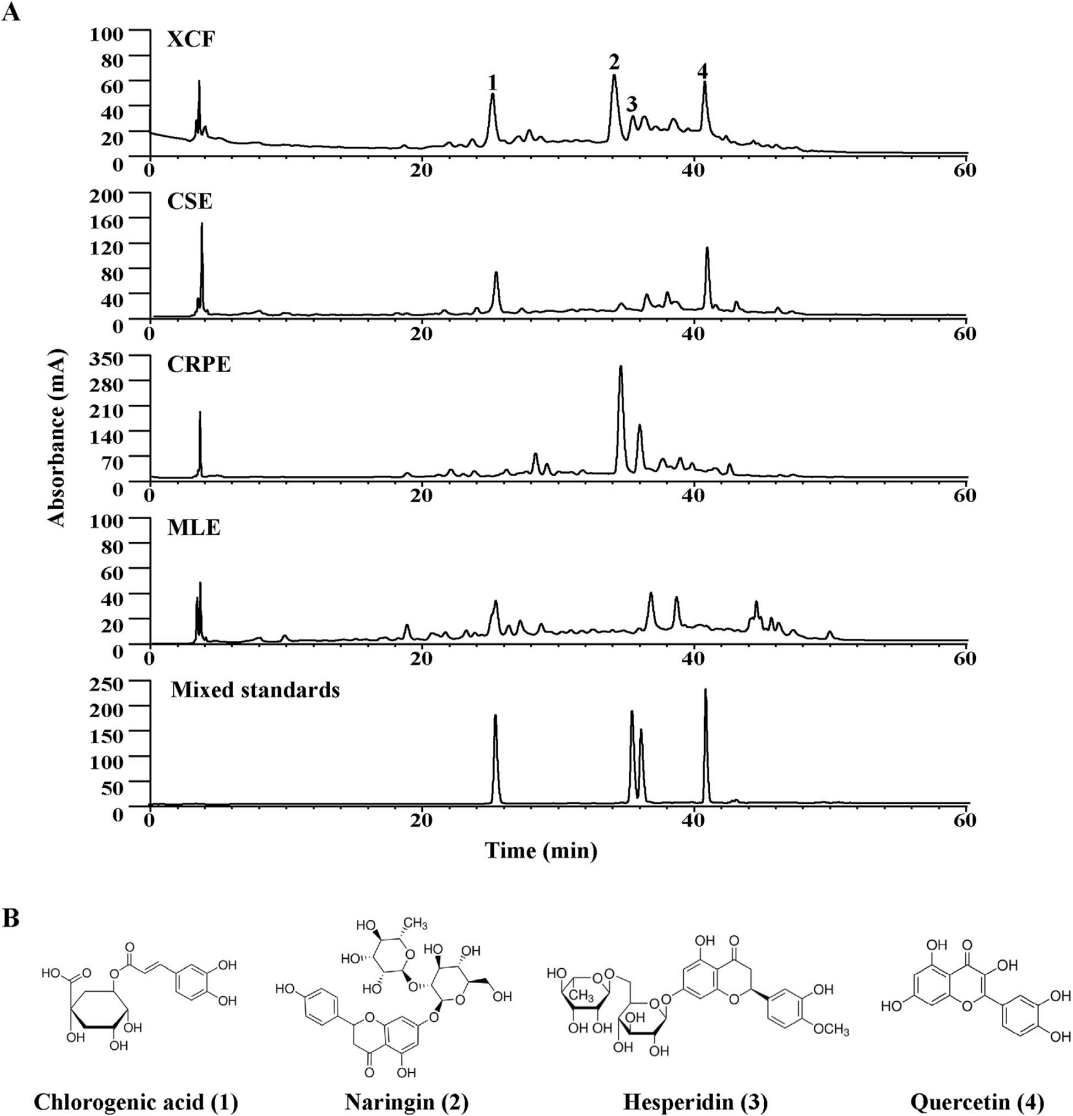
Chemical analysis of XCF by HPLC. **(A)** HPLC‒UV chromatograms of XCF, CSE, CRPE, MLE and mixed standards at 276 nm; **(B)** Structures of the four main chemical constituents in XCF. 1. Chlorogenic acid; 2. Naringin; 3. Hesperidin; 4. Quercetin.

The XCF and its composition herbs (namely, *C. setosum* and *M. alba* leaves and *C. reticulata* pericarpium) were simultaneously analyzed using the chromatographic fingerprinting analysis method. Peaks were assigned to each herb by comparing to the retention time of the peaks in the chromatograms of the individual herbal compouds. Four main peaks were unambiguously identified as chlorogenic acid (which were derived from CSE and MLE), naringin (which were derived from CRPE), hesperidin (which were derived from CRPE) and quercetin (which were derived from CSE) at 276 nm (Figures 3A, B) by comparing their retention times and UV spectra with those of commercial standard compounds. Chemical analysis of XCF and its composition herbs by HPLC at 254 nm as shown in Supplementary Figure S1. Chemical analysis of XCF by HPLC at 254 nm. Moreover, the contents of the four main active compounds in XCF were determined to be 2.71%, 0.92%, 0.40% and 1.47%, respectively, on the basis of their calibration curves, which were established by plotting the peak area against the standard substance concentration. The specific marking and testing ranges are shown in Supplementary Table S3. In addition, the distribution of the four compounds in the tested XCF batch is very similar. Chemical analysis of 3 batches of XCF by HPLC at 312/276/254 nm as shown in Supplementary Figures S2–S4.

### 3.4 XCF ameliorates lipid accumulation in mice with MAFLD

**FIGURE 4:**
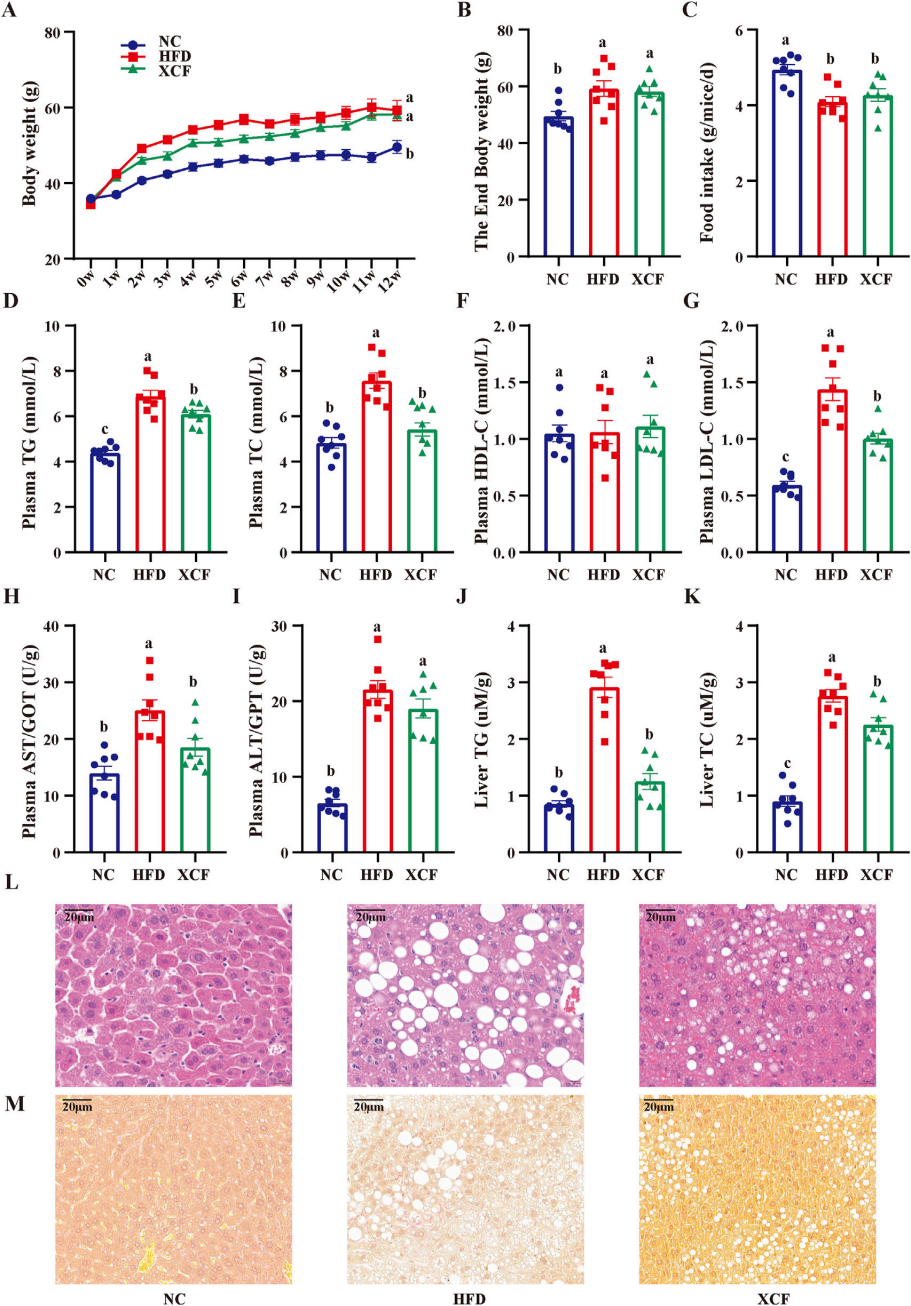
Effects of XCF on body weight and lipid accumulation in mice with MAFLD. **(A)** Body weight; **(B)** End body weight; **(C)** Food intake; Plasma TG **(D)**, TC **(E)**, HDL-C **(F)**, LDL-C **(G)** contents; The enzymatic activity of plasma AST/GOT **(H)**, ALT/GPT **(I)**; Liver TG **(J)**, TC **(K)** contents; **(L)** Micrographs of liver H&E staining; **(M)** Micrographs of liver Sirius red staining (bar = 20 μm). The data are presented as the means ± SEMs (n = 8). Data with different letters are significantly different (P < 0.05). NC: normal control group; HFD: high-fat diet group; XCF: xiaoji-chenpi formula extract treatment group. MAFLD, metabolic-associated fatty liver disease; TG, triglyceride; TC, total cholesterol; HDL-C, high-density lipoprotein cholesterol; LDL-C, low-density lipoprotein cholesterol; AST/GOT, aspartate aminotransferase/glutamic-oxal(o)acetic transaminase; ALT/GPT, alanine aminotransferase/glutamic-pyruvic transaminase; H&E, hematoxylin and eosin.

After feeding for 12 weeks, a significant increase (P < 0.05) in body weight was observed in the HFD group compared with the NC group (Figures 4A, B). The body weight of the XCF group was greater than that of the HFD group; however, the difference was not significant. Compared with that in the NC group, the food intake in the HFD group was lower (P < 0.05; Figure 4C) because of the higher energy content in the HFD group. Treatment with the XCF extract did not cause increased food intake compared with that in the HFD group. Furthermore, compared with those in the NC group, the plasma levels of TG, TC, and LDL-C were significantly increased (P < 0.05) in the HFD group (P < 0.05; Figures 4D–G), and XCF treatment significantly reversed these changes in TG and LDL-C. There was also a decrease in plasma TC in the XCF group compared with the HFD group, but this difference was not significant. There were no significant differences in the plasma HDL-C levels among the three groups. After HFD and XCF were administered for 12 weeks, significant increases in plasma AST and ALT (P < 0.05) were observed in the HFD group compared with the NC group. Treatment with XCF significantly decreased the levels of AST (P < 0.05, Figures 4H, I). However, there was no significant change in the plasma ALT level after XCF treatment. Moreover, the liver TC and TG levels were significantly increased (P < 0.05) in the HFD group but were significantly decreased by XCF treatment (P < 0.05, Figures 4J, K).

As shown in Figure 4L, robust lipid accumulation in hepatocytes was observed in the HFD group, indicating that HFD-fed mice exhibited MAFLD. Moreover, XCF treatment significantly ameliorated hepatic lipid accumulation. The number of lipid droplets was decreased, and the structure of hepatic cells was improved by XCF treatment, which indicates that XCF treatment alleviated the MAFLD caused by HFD. In addition, the liver was stained with Sirius red. Compared with the NC group, the HFD group presented significant bullous steatosis, collagen deposition, and a hepatic inflammatory state, as well as hepatic stripe collagen deposition and fibrosis. Compared with the HFD group, XCF treatment significantly reduced the inflammatory area, improved liver fibrosis, and restored the cell morphology (Figure 4M).

### 3.5 XCF treatment alters the transcriptomic profile of MAFLD model mice

**FIGURE 5:**
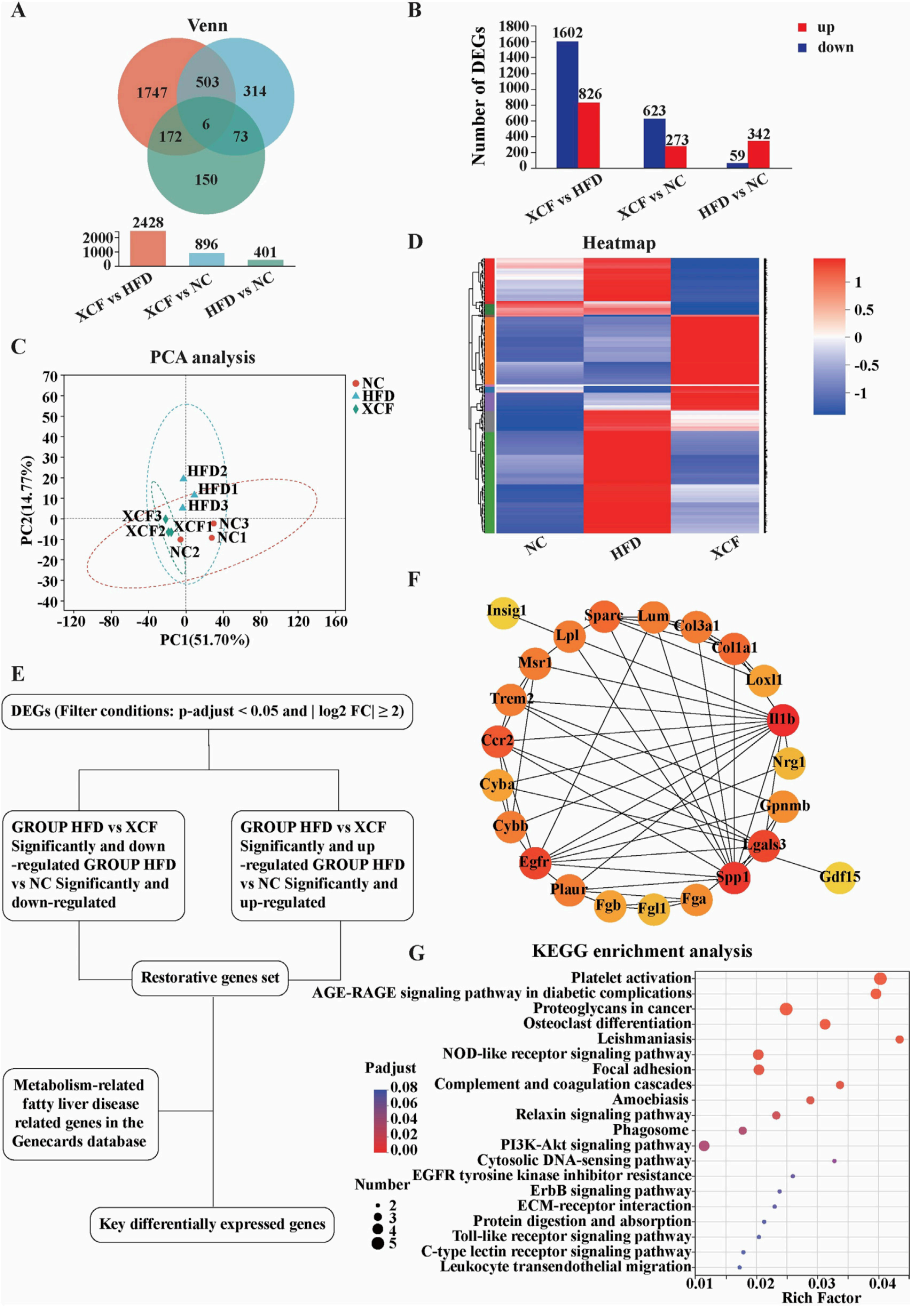
XCF treatment altered the transcriptomic profile of the mouse model. **(A)** Venn diagram; **(B)** Differential gene quantity statistics; **(C)** PCA; **(D)** Heatmap of differential gene clustering; **(E)** Schematic diagram of the key differential gene screening process; **(F)** Protein‒protein interaction network; **(G)** KEGG enrichment analysis. NC: normal control group; HFD: high-fat diet group; XCF: xiaoji-chenpi formula extract treatment group.

To investigate the potential mechanism by which XCF alleviates MAFLD, we used mouse liver tissues for RNA-seq research. A total of 2,965 differentially expressed genes were identified via RESM software (P-adjust < 0.05 and | log_2_FC | ≥ 2) in three pairwise comparisons: XCF vs HFD, XCF vs. NC and HFD vs. NC. The results are shown with a Venn diagram. Compared with those in the HFD group, a total of 2,428 differentially expressed genes were detected in the XCF group, including 1602 downregulated genes and 826 upregulated genes. Compared with those in the NC group, a total of 896 differentially expressed genes were detected in the XCF group, including 623 downregulated genes and 273 upregulated genes. Compared with those in the NC group, a total of 401 differentially expressed genes were detected in the HFD group, including 59 downregulated genes and 342 upregulated genes (Figures 5A, B). The PCA results shown in Figure 5C revealed that the gene expression in the HFD group deviated from that in the NC group, and the gene expression in the XCF group was close to that in the NC group. Hierarchical clustering was used to analyze the gene expression profiles, revealing that the trend of gene expression in the XCF group was more similar to that in the NC group than it was in the HFD group (Figure 5D). A total of 175 restorative genes were identified, as shown in the flow chart in Figure 5E. Compared with those in the HFD group, 152 genes in the XCF group were significantly reduced to levels similar to those in the NC group, and 23 genes in the XCF group were significantly increased to levels similar to those in the NC group.

To search for key genes related to MAFLD in the liver transcriptome after XCF treatment, we downloaded the gene sets in the GeneCards public database with the keywords ‘NAFLD’ and ‘MAFLD’. Using Venn2.1 software, 31 intersecting genes were obtained from the public database gene set and transcriptome restoration genes. The PPI network was generated via the STRING online database, as shown in Figures 5E, F. The nodes and edges in the network represent the target protein and protein‒protein associations, respectively. We identified hub targets by the degree size of nodes. IL-1β is the most critical hub target in this network, followed by SPP1 and LGALS3. These protein targets with high degree values in the network may be the main mechanism by which XCF treats MAFLD. Furthermore, the results of the KEGG pathway enrichment analysis are shown in Figure 5G (the top 20 pathways). “Platelet activation” and “AGE-RAGE signaling pathway in diabetic complications” were the most significant pathways.

### 3.6 Effects of XCF on lipid synthesis metabolism, fibrosis, and inflammation-related gene expression in the livers of MAFLD mice

**FIGURE 6:**
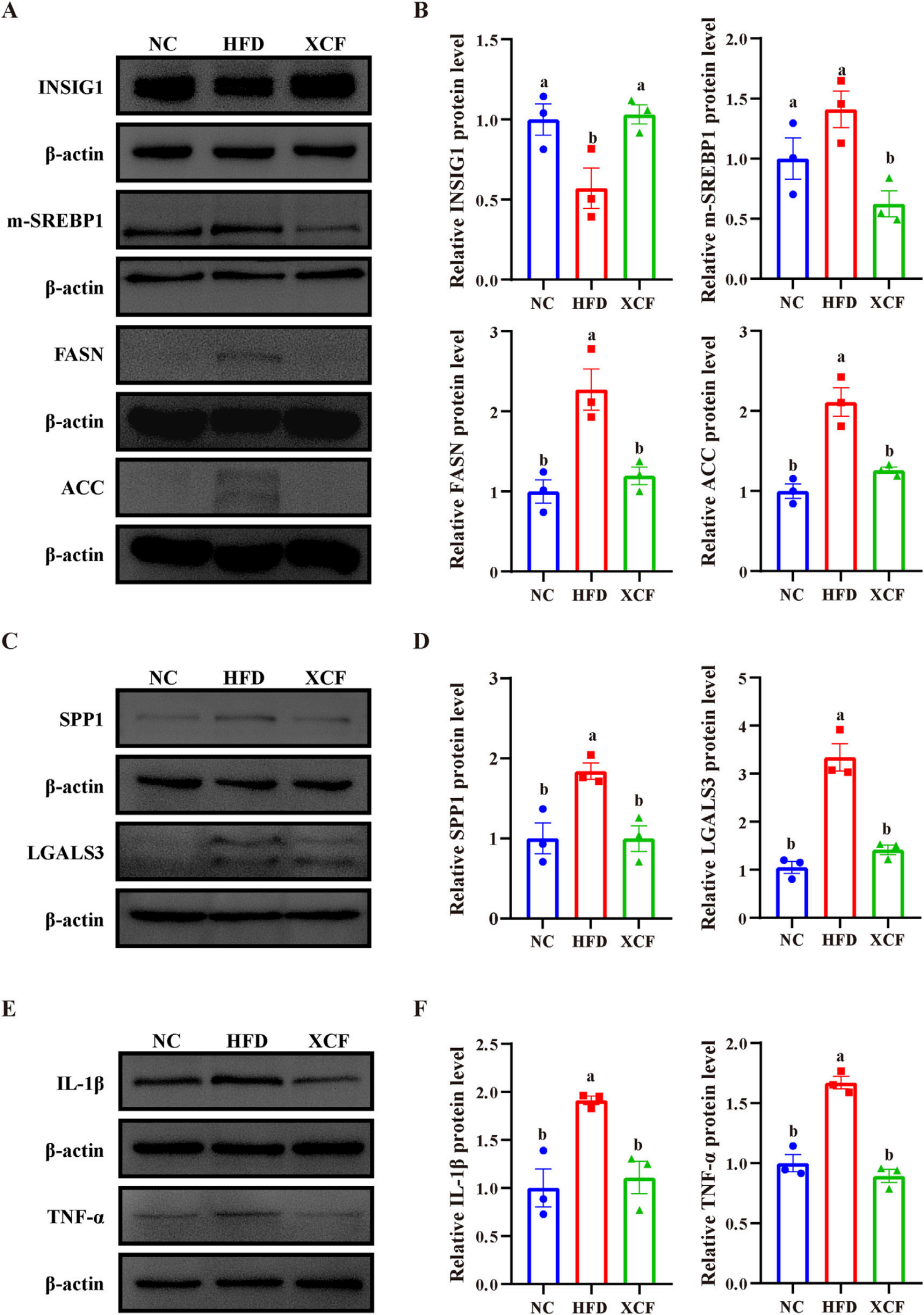
Effects of XCF on lipid synthesis metabolism, fibrosis, and inflammation-related gene expression in the livers of HFD-fed mice. **(A, B)** Relative protein expression of INSIG1, m-SREBP1, FASN and ACC in the liver. **(C, D)** Relative protein expression of SPP1 and LGALS3 in the liver. **(E, F)** Relative protein expression of IL-1β and TNF-α in the liver. Data are presented as the means ± SEMs (n = 3). Data with different letters are significantly different (P < 0.05). NC: normal control group; HFD: high-fat diet group; XCF: xiaoji-chenpi formula extract treatment group.

The INSIG1/SREBP1 pathway plays a crucial role in lipid homeostasis, and fibrosis and inflammation collectively facilitate the progression of MAFLD. To investigate the underlying mechanisms of action of XCF against MAFLD, we performed experimental validation of the key differentially expressed genes. As shown in Figures 6A, B, the protein level of INSIG1 in HFD-induced MAFLD mice was significantly lower than that in control mice, whereas the expression of INSIG1 in XCF mice was significantly increased. In addition, the protein levels of m-SREBP1c, FASN and ACC in HFD-fed mice also increased, and XCF treatment effectively reduced these protein levels (P < 0.05). These results indicated that XCF treatment inhibited the synthesis of fatty acids in the mice. Compared with the NC group, the HFD group presented significantly increased protein levels of SPP1 and LGALS3, which were reversed by XCF treatment and returned to normal levels (P < 0.05, Figures 6C, D). In addition, similar changes in the protein levels of inflammatory factors were observed. The protein levels of IL-1β and TNF-α were significantly increased in the HFD-fed mice, and XCF treatment reversed these changes, which suggested that XCF treatment can ameliorate inflammation in HFD-fed mice (P < 0.05, Figures 6E, F). These results indicate that XCF modulates lipid metabolism via the INSIG1/SREBP1 pathway *in vivo*, improves MAFLD-related fibrosis via SPP1 and LGALS3 and inhibits inflammation in MAFLD.

## 4 Discussion

Considering the impact of MAFLD on the economy and on patients’ quality of life, there is an urgent need to find safe and effective natural medicines to prevent this disease. TCM formulas are based on various active compounds and act on multiple targets to exert their pharmacological activity, indicating their unique clinical and scientific value (Atanasov et al., 2015). Clinical trials and our previous research have shown that QGS can improve MAFLD with fewer adverse reactions and good therapeutic pharmacological activity (Yang et al., 2021). The application of TCM theory to optimize formulas is prevalent, and XCF and XJF, which are disassembled formulas derived from QGS, are predicted to have potential for treating MAFLD. In our study, we systematically investigated the feeding conditions required for establishing female zebrafish MAFLD model suitable for high-throughput screening of various formulations, with feed containing 4% cholesterol and 20% fat content. In addition, we successfully compared the differences in anti-MAFLD activity between XCF and XJF and found that XCF had the best pharmacological activity. Four main active compounds in XCF were identified via high-performance liquid chromatography: chlorogenic acid, quercetin, naringin, and hesperidin, which were derived from *C. setosum*, *M. alba* leaves, and *C. reticulata* pericarpium, respectively. By utilizing a HFD mouse model and RNA sequencing, we elucidated the pharmacological effects and molecular mechanisms of XCF in mitigating MAFLD. Specifically, XCF was found to improve lipid metabolism, inhibit inflammation, and reduce liver fibrosis in mice with MAFLD.

Given the complex pathogenesis of MAFLD, tissue, organ, and cellular levels cannot reflect the overall disease status. Using traditional animal models to screen multiple TCM formulas is a more effective method. Mammalian models such as pigs, rabbits, and mice have the advantages of mature experimental methods, good modeling stability, and convenient sampling. However, their disadvantages, such as long modeling times and high feeding costs, hinder in-depth research on MAFLD in mammalian models. Therefore, developing new animal models for MAFLD research is highly challenging. Small model organisms such as *D. melanogaster*, *Caenorhabditis elegans*, and zebrafish have also been gradually developed for MAFLD research (Lo et al., 2012; Musselman and Kühnlein, 2018; Gut et al., 2017). However, for *Drosophila melanogaster* and *C. elegans*, it may not be possible to simulate human lipid pathway metabolism effectively. Thus, it is difficult to directly apply the pathological changes observed in worms to the diagnosis and treatment of human MAFLD. Zebrafish, as a model organism with a genome sequence that shares approximately 70% similarity with that of humans, has been widely used in research on liver metabolism-related diseases such as hyperlipidemia, nonalcoholic fatty liver disease, and liver fibrosis in recent years because its blood lipid composition, lipid absorption, and lipid metabolism processes are essentially identical to those of humans (Howe et al., 2013; Gut et al., 2017). Owing to their similarity with humans in terms of liver cell composition, function, signaling, and response to injury, zebrafish have become powerful models for screening lipid-lowering and hepatoprotective drugs and studying the pathogenesis of liver disease (Shimizu et al., 2023). However, there are currently certain differences in the research design of zebrafish MAFLD models, and a relatively standardized method has yet to be established. Most single modeling methods have inherent limitations in the research process. For example, excessive feeding of egg yolks and other ingredients may lead to batch differences due to uneven distribution (Silva et al., 2024). Consequently, the composite model, which combines dietary manipulation with gene defects, has emerged as a promising research topic. This approach can potentially reduce the modeling time and improve the success rate of the model. Nevertheless, gene editing remains a challenging and costly process, rendering it impractical for large-scale screening purposes (Li et al., 2021). In our study, we conducted an orthogonal experiment to explore the optimal feeding concentrations of fat and cholesterol in standardized feed. The feasibility of inducing a zebrafish MAFLD model with a 4% cholesterol and 20% fat diet was verified by measuring the zebrafish BMI and performing liver H&E staining. Owing to the strict regulation of liver lipid metabolism by multiple interrelated genes, we also used RNA-seq to identify DEGs and explore key pathways affecting the liver under high-fat and high-cholesterol diets. The results of the KEGG analysis of the liver transcriptome revealed that it was enriched mainly in the steroid biosynthesis and glycosylgolipid biosynthesis lacto- and neolacto-series pathways. Core lipid metabolism-related genes, such as methylsterol monooxygenase 1 (MSMO1), transmembrane seven superfamily member 2 (TM7SF2), sterol-c5-desaturase (SC5D), IMP U3 small nucleolar ribonucleoprotein 3 (IMP3), RNA polymerase I subunit D (POLR1D) and solute carrier family one member 1 (SLC1A1), were revealed in the PPI. The results of the KEGG pathway and PPI analyses revealed the mechanism of lipid synthesis-related pathways, which still needs to be further validated through combined experiments in the future. In subsequent traditional Chinese medicine prescription screening experiments, we also established resveratrol and silymarin treatment groups as positive drug controls, as these two drugs have been shown to be beneficial to MAFLD (Izzo et al., 2021; Gillessen and Schmidt, 2020; Huang et al., 2024). Resveratrol and silymarin treatment improves liver steatosis in zebrafish with MAFLD. These results highlight the novel ability of zebrafish to model the pathogenesis of MAFLD and validate their feasibility for *in vivo* screening of novel pharmacological interventions. A comparison of the pharmacological activities of the formulas revealed that XCF was more effective than XJF at improving liver lesions in the zebrafish MAFLD model. Additionally, it is noteworthy that sex may play a pivotal role in establishing MAFLD models in zebrafish. Prior studies have shown that under identical diet-induced obesity conditions, female zebrafish exhibit more pronounced changes in body weight and BMI compared to males (Newman et al., 2016; Navarro-Barrón et al., 2019). Hence, we selected female zebrafish for this study. Although our results indicated that a diet with 4% cholesterol and 20% fat effectively induced MAFLD in female zebrafish and reveals mechanisms related to lipid synthesis pathways, these conclusions may not fully apply to males. Given the significant physiological, metabolic, and behavioral differences between male and female zebrafish, these disparities may affect their responses to high-fat and high-cholesterol diets. Studies have shown that excess high-fat diets promote sex-specific alterations in zebrafish gut microbiota (Navarro-Barrón et al., 2019), and Oka et al. observed sex differences in the lipid profile of zebrafish (Oka et al., 2010). Therefore, future studies should further explore the impact of sex on MAFLD model establishment in zebrafish to ensure model accuracy and reliability.

A HFD can lead to excessive deposition of lipids in the liver. Research has shown that the INSIG1/SREBP1 pathway is a key pathway involved in regulating cellular lipid metabolism and is closely related to the occurrence of MAFLD (Carobbio et al., 2013; Wang Fuchun et al., 2024). INSIG, a protein that is attached to the ER and is an important regulator of cholesterol and fat synthesis, plays a crucial role in maintaining cellular lipid homeostasis. It has two subtypes: insulin-induced gene 1 (INSIG1) and insulin-induced gene 2 (INSIG2). INSIG1 is expressed mainly in the liver and anchors the key transcription factor sterol regulatory element-binding protein 1 (SREBP1), which regulates lipid metabolism to the endoplasmic reticulum, reducing the splicing of Golgi transport and the cleavage of SREBP1 into the mature form (m-SREBP1) into the nucleus and the expression of its target genes (Cheng et al., 2022). SREBP1, is a member of the SREBP family, is primarily responsible for regulating the synthesis of fatty acids and triglycerides. Fatty acid synthase (FASN) and acetyl-CoA carboxylase (ACC) are downstream target genes of SREBP1 (Xu et al., 2024). FASN is a key enzyme in fatty acid synthesis that catalyzes acetyl-CoA, which is used in fatty acid synthesis. Previous studies have shown that FASN overexpression promotes the accumulation of lipids in liver cells and induces oxidative damage to liver tissue, leading to lipid and energy metabolism disorders (Lan et al., 2024). ACC also plays an important role in liver lipid metabolism, catalyzing the formation of malonyl CoA from CoA and participating in the regulation of fatty acid synthesis and oxidation processes in the liver (McGarry et al., 1977). We used transcriptomics techniques to analyze mouse liver samples and screen for differentially expressed genes related to lipid metabolism after XCF treatment. INSIG1 was identified as a key gene and its downstream gene pathway SREBP1/FASN/ACC. Western blot analysis revealed that the relative protein levels of INSIG1 decreased in the HFD group, whereas the relative protein levels of m-SREBP1, FASN, and ACC increased. XCF treatment resulted in an increase in INSIG1 levels and a decrease in m-SREBP1, FASN, and ACC levels. These results indicate that XCF has a significant lipid-lowering effect. XCF may alleviate MAFLD by increasing INSIG1 and reducing the relative protein levels of m-SREBP1, FASN, and ACC, thereby reducing lipid accumulation. In this study, we identified four main compounds in XCF. Chlorogenic acid was one of the main compound of *C. setosum* and *M. alba* leaves. Naringin and hesperidin were the main compounds of *C. reticulata* pericarpium. Quercetin was the main compound of *C. setosum*. According to previous studies, naringin efficiently promotes hepatic *de novo* fatty acid synthesis by downregulating the protein expression of SREBP1, FAS, ACC, and stearoyl-CoA desaturase 1 (SCD1) in HFD-fed mice (Mu et al., 2020). Hesperidin, chlorogenic acid, and quercetin significantly reduced SREBP1 and FAS gene expression in an *in vitro* model (Gómez-Zorita et al., 2017; Liu et al., 2020; Mosqueda-Solís et al., 2017). These findings also support our experimental results.

Inflammation is one of the key driving factors for the progression of simple fatty liver disease to steatohepatitis (Peiseler and Frank, 2021). The release of proinflammatory cytokines, such as IL-1β and TNF-α, can induce liver injury. When liver injury occurs, the serum transaminase levels (including ALT and AST) significantly increase (Kim et al., 2008; McGill, 2016). The protein encoded by IL-1β is a member of the interleukin-1 cytokine family. IL-1β is a potent proinflammatory cytokine that is produced primarily by monocytes and macrophages (Gupta and Barthwal, 2018). Our study revealed that XCF treatment significantly reduced ALT and AST levels in MAFLD mice fed a HFD. In addition, western blot analysis revealed that XCF treatment led to a decrease in the protein levels of IL-1β and TNF-α in liver tissue. These results indicate that XCF may reduce liver inflammation and damage by decreasing the relative protein levels of IL-1β and TNF-α, thereby alleviating MAFLD. In addition, four main compounds have been proven to have anti-inflammatory cytokine-lowering pharmacological activity both *in vitro* and *in vivo* (El-Desoky et al., 2018; Tejada et al., 2018; Zhang et al., 2022; Huang et al., 2023), providing support for current research on the anti-inflammatory activity of XCF in MAFLD mice.

Nonalcoholic steatohepatitis (NASH) is the progressive form of MAFLD (Kumar et al., 2021). NASH involves severe hepatocyte injury and liver inflammation. Therefore, liver fibrosis and inflammation are also key to disease treatment. The activity of liver stellate cells can promote the excretion of ECM, pro-inflammatory fine cell factor and protease, which further induce cell damage and inflammation (Wang Chao et al., 2022). The galectin-3 (Gal-3/LGALS3) protein, an S-type lectin, is encoded by the galectin-3 gene. It includes a carbohydrate recognition domain (CRD), collagen-like tandem repeats of nine amino acids and an N-terminal 12-mer peptide (Krześlak and Lipińska, 2004). LGALS3 plays an important role in MAFLD and its progression to more severe liver disease states, such as liver fibrosis and cirrhosis. Studies have shown that the overexpression of LGALS3 is closely associated with poor prognosis and tumor immune cell infiltration in hepatocellular carcinoma (HCC). Studies have also indicated that cholesterol induces a smooth muscle cell (SMC) phenotype transition, which features high LGALS3 expression (Rong et al., 2003). As the master regulator of lipid metabolism, SREBP1 positively regulates LGALS3 expression and *vice versa* (Li J. et al., 2022). OPN/SPP1 is a glycosylated protein that is widely present in the extracellular matrix and is closely associated with liver TG in NAFLD patients. SPP1, a profibrotic factor, plays a crucial role in NASH and liver fibrosis processes (Song et al., 2021). During the course of NASH, stressed and dead liver parenchymal cells release profibrotic factors such as SPP1, which further recruit and activate liver macrophages and hepatic stellate cells, thereby exacerbating the process of liver fibrosis (Han et al., 2023). Our findings indicate that XCF can significantly reduce the mRNA and protein expression levels of LGALS3 and SPP1 in liver tissue induced by a HFD, thereby alleviating the degree of liver fibrosis in MAFLD mice. These results are also supported by previous studies. The molecular docking results demonstrated that quercetin strongly bound to the SPP1 protein (Wang Man et al., 2022). Naringin inhibited inflammation to relieve liver fibrosis through the TGF-β-Smad signaling pathway (Wang Lechen et al., 2024). Additionally, studies have shown that hesperidin and chlorogenic acid can inhibit the activation of hepatic stellate cells (Elshazly and Amr, 2014; Miao et al., 2022). However, further investigations are needed to determine whether SPP1 and LGALS3 are direct binding targets of these four compounds.

Currently, the therapeutic strategies for MAFLD primarily focus on lifestyle modifications, such as improving dietary patterns and increasing physical activity, complemented by pharmacological interventions aimed at enhancing insulin sensitivity and reducing hepatic inflammation. In terms of pharmacological treatment for MAFLD and its severe form, MASH, Rezdiffra stands alone as the only drug approved by the U.S. Food and Drug Administration (FDA) specifically for the treatment of MASH (Harrison et al., 2024). Current adjuvant therapeutic medications predominantly target the alleviation of liver damage and extrahepatic complications, encompassing a variety of categories including insulin sensitizers, lipid-lowering agents, and antioxidants. For instance, metformin, the preferred medication for the management of Type 2 Diabetes Mellitus (T2DM) (American Diabetes Association, 2019), has been proven to exert a positive therapeutic effect on MAFLD by activating the AMPK/INSIG1 signaling pathway, which subsequently inhibits the maturation of SREBP1 and the process of lipid synthesis (Han et al., 2019). Our research findings have unveiled the potential mechanism of pharmacological activity of XCF in MAFLD, involving multiple regulatory pathways related to lipid metabolism, inflammatory response, and fibrotic processes. XCF demonstrates the ability to modulate the INSIG1/SREBP1 pathway and its downstream targets FASN and ACC, suggesting its potential for combined use with drugs targeting these pathways to further enhance therapeutic efficacy. Similarly noteworthy is the anti-inflammatory and anti-fibrotic properties of XCF, which may exhibit synergistic effects when used in conjunction with medications aimed at reducing hepatic inflammation and fibrosis. Furthermore, compounds such as chlorogenic acid, naringin, hesperidin, and quercetin, serving as effective active ingredients in XCF for the treatment of MAFLD, possess tremendous potential and merit further in-depth investigation. In future experiments, we will conduct a thorough analysis of the synergistic pharmacological activities among the components within XCF and explore the specific impacts of individual compounds or various combinations of compounds in conjunction with clinical medications on MAFLD, with the aim of providing more scientific evidence for the treatment of this disease.

Although RNA sequencing is a powerful tool for gene expression analysis, it also has limitations such as instability, high false positive rates, and inability to directly detect protein posttranslational modifications (González and Simon, 2013). In practical applications, it is necessary to comprehensively consider these factors and combine them with other technical means to improve the accuracy and comprehensiveness of research. In addition to lipid synthesis, inflammation, and fibrosis pathways, XCF may lead to the weakening of MAFLD through different mechanisms, such as imbalanced gut microbiota. The exact pharmacological mechanism of XCF in treating NAFLD should be explored in the future. Therefore, in clinical applications, these limitations need to be fully considered, individualized treatment should be administered on the basis of the specific situation of patients, and relevant research and quality control work should be strengthened to improve the pharmacological activity and safety of traditional Chinese medicine therapy for MAFLD.

## 5 Conclusion

In conclusion,we systematically established female zebrafish MAFLD model for high-throughput screening and compared the pharmacological activity of XCF versus XJF. Using HPLC, we identified key active compounds in XCF. Through an HFD mouse model and RNA-seq, XCF improved lipid metabolism, inhibited inflammation, and reduced liver fibrosis in MAFLD. This study can better explain the mechanism basis of XCF improving MAFLD as a whole Additionally, the multilevel validation process established by our research institute serves as a valuable reference for future investigations into the comparative activity of diverse and complex TCM against MAFLD.

## Data Availability

The datasets presented in this study can be found in online repositories. The names of the repository/repositories and accession number(s) can be found in the article/Supplementary Material.

## Glossary

XCF: Xiaoji-chenpi formula
MAFLD: Metabolic-associated fatty liver disease
QGS: QingGanSan
BMI: Body mass index
HFD: High-fat diet
HPLC: High-performance liquid chromatography
TC: Total cholesterol
TG: Triglycerides
INSIG1/2: Insulin-induced gene 1/2
SREBP1: Sterol regulatory element-binding protein-1
FASN: Fatty acid synthase
ACC: Acetyl-CoA carboxylase
SPP1/OPN: Secreted phosphoprotein 1/osteopontin
LGALS3/Gal-3: Lectin galactoside-binding soluble 3/galectin-3
TNF-α: Tumor necrosis factor-alpha
IL-1β: Interleukin-1beta
NAFLD: Nonalcoholic fatty liver disease
TCM: Traditional Chinese medicine
XJF: Xiaoji-jujv formula
LDL-C: Low-density lipoprotein cholesterol
HDL-C: High-density lipoprotein cholesterol
NC: Normal control
HFD: High-fat diet
HFCD: High-fat and high-fat cholesterol diet
ALT: Alanine aminotransferase
AST: Aspartate aminotransferase
CSE: *C. setosum* extract
CRPE: *C. reticulata* pericarpium extract
MLE: *M. alba* leaves extract
H&E: Haematoxylin and eosin
DEGs: Differentially expressed genes
PPI: Protein-protein interaction
KEGG: Kyoto Encyclopedia of Genes and Genomes
ECL: Enhanced chemiluminescence
SEMs: Standard errors of the means
NASH: Non-alcoholic steatohepatitis
T2DM: Type 2 diabetes mellitus
