# Supplementary material for: An integrative pharmacology-based study on the pharmacological activity and mechanism of xiaoji-chenpi formula (XCF) against MAFLD
Source: Front Pharmacol. 2025 Mar 7;16:1521111. doi: 10.3389/fphar.2025.1521111 (PMC11925881; doi:10.3389/fphar.2025.1521111)
Supplement: Supplementary file 3 [file DataSheet1.docx]

Supplementary Material

# Supplementary Figures

## Supplementary Figure S1-S4

**Supplementary Figure S1.** Chemical analysis of XCF by HPLC at 254 nm. (A) HPLC‒UV chromatograms of XCF, CSE, CRPE, MLE and mixed standards at 254 nm.

**Supplementary Figure S2.** Chemical analysis of 3 batches of XCF by HPLC at 312 nm. (A) HPLC fingerprint of 3 batches of XCF at 312 nm; (B) HPLC fingerprint of chlorogenic acid, naringin, hesperidin, and quercetin at 312 nm.

**Supplementary Figure S3.** Chemical analysis of 3 batches of XCF by HPLC at 276 nm. (A) HPLC fingerprint of 3 batches of XCF at 276 nm; (B) HPLC fingerprint of chlorogenic acid, naringin, hesperidin, and quercetin at 276 nm.

**Supplementary Figure S4.** Chemical analysis of 3 batches of XCF by HPLC at 254 nm. (A) HPLC fingerprint of 3 batches of XCF at 254 nm; (B) HPLC fingerprint of chlorogenic acid, naringin, hesperidin, and quercetin at 254 nm.

## Supplementary Figure S5

**Supplementary Figure S5.** The experimental grouping of three batches of zebrafish and the experimental sequence are as follows: Experiment 1; Experiment 2; Experiment 3
